# A docking study of enhanced intracellular survival protein from *Mycobacterium tuberculosis* with human DUSP16/MKP-7

**DOI:** 10.1107/S0909049513021341

**Published:** 2013-10-01

**Authors:** Hye-Jin Yoon, Kyoung Hoon Kim, Jin Kuk Yang, Se Won Suh, Hyunsik Kim, Soonmin Jang

**Affiliations:** a Department of Chemistry, College of Natural Sciences, Seoul National University, Seoul 151-747, Republic of Korea; b Department of Chemistry, College of Natural Sciences, Soongsil University, Seoul 156-743, Republic of Korea; c Department of Biophysics and Chemical Biology, College of Natural Sciences, Seoul National University, Seoul 151-747, Republic of Korea; d Department of Chemistry, College of Natural Sciences, Sejong University, Seoul 143-747, Republic of Korea

**Keywords:** protein–protein docking, Eis, *Mycobacterium tuberculosis*, *HADDOCK*, comparative docking, docking study

## Abstract

The intracellular pathogen *Mycobacterium tuberculosis* (*Mtb*) causes tuberculosis, and one of its secreted effector proteins, called enhanced intracellular survival (Eis) protein, enhances its survival in macrophages. *Mtb* Eis activates JNK-specific dual-specificity protein phosphatase 16 (DUSP16)/mitogen-activated protein kinase phosphatase-7 (MKP-7) through the acetylation on Lys55, thus inactivating JNK by dephosphorylation. Based on the recently reported crystal structure of *Mtb* Eis, a docking model for the binding of *Mtb* Eis to DUSP16/MKP-7 was generated. In the docking model, the substrate helix containing Lys55 of DUSP16/MKP-7 fits nicely into the active-site cleft of *Mtb* Eis; the twisted β-sheet of Eis domain II embraces the substrate helix from one side. Most importantly, the side-chain of Lys55 is inserted toward acetyl-CoA and the resulting distance is 4.6 Å between the NZ atom of Lys55 and the carbonyl carbon of the acetyl group in acetyl-CoA. The binding of *Mtb* Eis and DUSP16/MKP-7 is maintained by strong electrostatic interactions. The active-site cleft of *Mtb* Eis has a negatively charged surface formed by Asp25, Glu138, Asp286, Glu395 and the terminal carboxylic group of Phe396. In contrast, DUSP16/MKP-7 contains five basic residues, Lys52, Lys55, Arg56, Arg57 and Lys62, which point toward the negatively charged surface of the active-site pocket of *Mtb* Eis. Thus, the current docking model suggests that the binding of DUSP16/MKP-7 to *Mtb* Eis should be established by charge complementarity in addition to a very favorable geometric arrangement. The suggested mode of binding requires the dissociation of the hexameric *Mtb* Eis into dimers or monomers. This study may be useful for future studies aiming to develop inhibitors of *Mtb* Eis as a new anti-tuberculosis drug candidate.

## Abstract 2

A docking study of *Mtb* Eis with its substrate DUSP16/MKP-7 was performed. The docking model suggests dissociation of hexameric *Mtb* Eis into dimers or monomers.

## 1. Introduction

Tuberculosis, caused by pathogenic bacterium *Mycobacterium tuberculosis* (*Mtb*), is difficult to treat and increasing drug-resistance poses a serious global health threat (Dye & Williams, 2010 ▶; Chiang *et al.*, 2010 ▶). This raises the urgent need for developing a new class of anti-tuberculosis drugs, which prompts the identification of novel drug target proteins of *Mtb*. Since one of survival strategies of *Mtb* is to inhibit the phagosomal maturation and autophagy in macrophages, *Mtb* effector proteins involved in these processes are of great interest for therapeutic intervention (Meena & Rajni, 2010 ▶). Eis (enhanced intracellular survival) protein secreted by *Mtb* was shown to enhance the survival of *M. smegmatis* (*Msm*) in the human macrophage-like cell line (Wei *et al.*, 2000 ▶). The survival enhancement by *Mtb* Eis is achieved by down-regulating the JNK (c-Jun N-terminal kinase), which leads to the inhibition of inflammation and autophagic cell death (Shin *et al.*, 2010 ▶). We recently revealed the molecular mechanism by which *Mtb* Eis down-regulates JNK and thereby enhances *Mtb* survival (Kim *et al.*, 2012 ▶). In our recent study, *Mtb* Eis was shown to be an efficient N^∊^-acetyltransferase acting on Lys55 of DUSP16/MKP-7 (Kim *et al.*, 2012 ▶), which is a JNK-specific phosphatase (Masuda *et al.*, 2001 ▶). *Mtb* Eis activates DUSP16/MKP-7 through the acetylation, and the activated phosphatase inactivates JNK by dephosphorylation (Kim *et al.*, 2012 ▶). The crystal structure determined in the same study revealed that the active-site cleft is formed in the interface between domains I and II (Kim *et al.*, 2012 ▶). More importantly, the active-site cleft has a negatively charged surface, which seems suitable to elicit the binding of the positively charged substrate, Lys55 of DUSP16/MKP-7 (Kim *et al.*, 2012 ▶).

To better understand the molecular details of the inter­action between *Mtb* Eis and its substrate, DUSP16/MKP-7, we carried out the molecular docking with previously determined crystal structures of the both proteins [Protein Data Bank (PDB) accession numbers 3ryo for *Mtb* Eis (Kim *et al.*, 2012 ▶) and 2vsw for DUSP16/MKP-7 (not published)]. The docked model suggests that the helix harboring Lys55 of DUSP16/MKP-7 fits into the active-site cleft of *Mtb* Eis and the binding may be established by not only the structural fit but also the electrostatic complementarity. Interestingly, however, the predicted binding mode requires dissociation of the hexameric *Mtb* Eis. This study may provide the structural basis for future research aiming to develop the inhibitor of *Mtb* Eis as a new tuberculosis drug.

## 2. Material and methods

We have used *HADDOCK* version 2.1 (de Vries *et al.*, 2007 ▶; van Dijk *et al.*, 2006 ▶) for the docking study. In *HADDOCK*, the overall docking is completed in three stages. It generates thousands of possible arrangements with starting structures and obtains hundreds of best structures using rigid-body docking for the next step. Then, these best structures are subject to semi-flexible docking, followed by water solvation. The semi-flexible docking employed in *HADDOCK* proceeds in four different stages as follows. The first and second stages are high-temperature rigid-body search and rigid-body simulated annealing (SA), respectively. The third and fourth stages are semi-flexible SA with flexible side-chains at the interface and semi-flexible SA with flexible backbone and side-chains, respectively. Here, the semi-flexible SA means SA in torsion-angle space. In this study, the values of the number of initial docked structure generations and the number of best structures from rigid-body docking were 2000 and 400, respectively. Among those 400 structures, we retained 200 best structures after the *HADDOCK* solvation analysis step.

**Figure 1:**
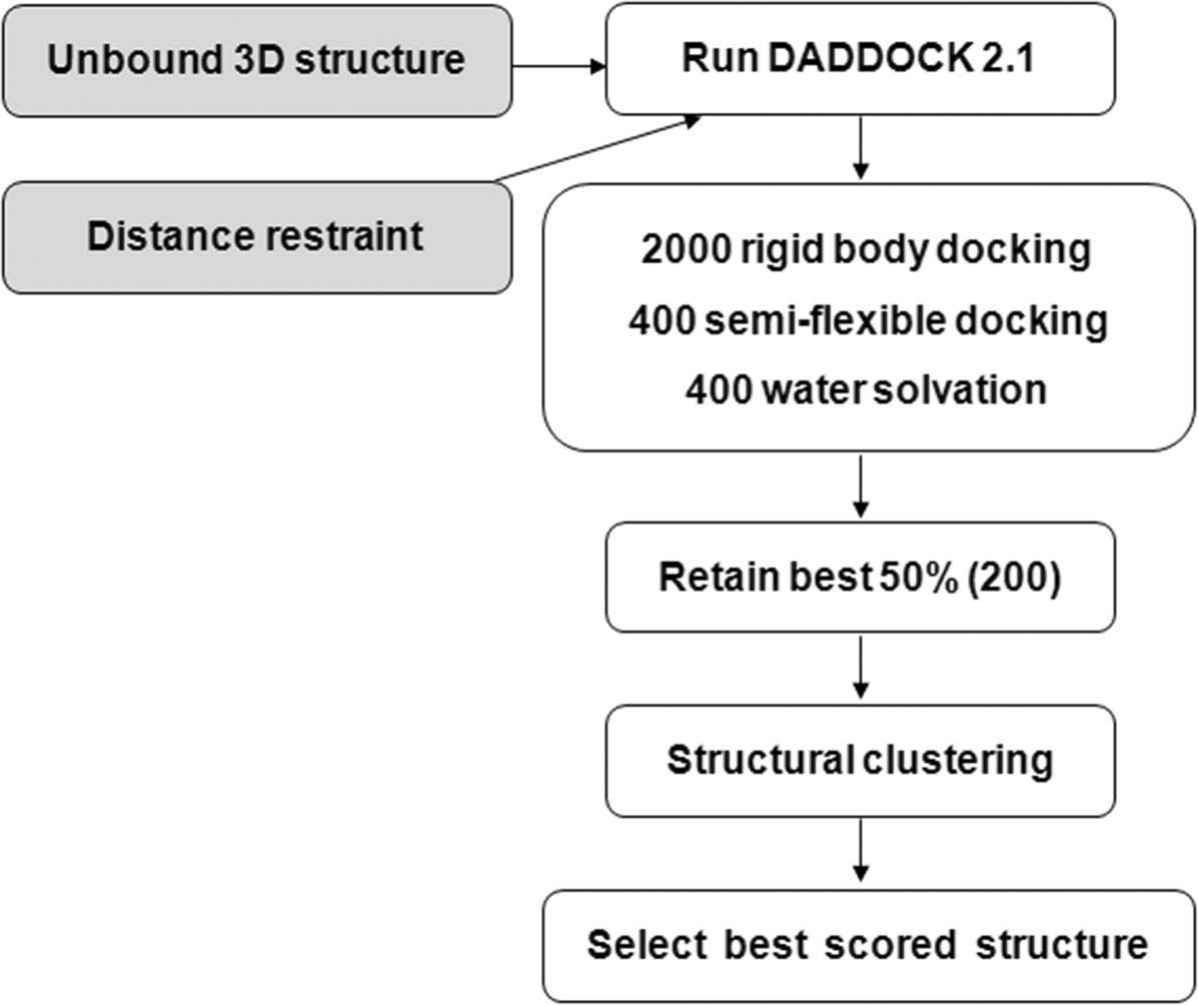
*HADDOCK* protocol flowchart employed in this study.

Other than the above changes, we have used the default *HADDOCK* parameters with version 5.4 of protein and solvent topologies throughout the docking procedure. The starting structure of *Mtb* Eis with acetyl-CoA was obtained from our previous study (PDB code: 3ryo) and the DUSP16/MKP-7 structure was taken from the PDB (unpublished; PDB ID 2vsw). This is a three-body docking problem but we docked DUSP16/MKP-7 with *Mtb* Eis in the presence of acetyl-CoA inside *Mtb* Eis, reducing it to a usual two-body docking task effectively. We performed two sets of independent docking simulations, *i.e.* docking of single DUSP16/MKP-7 with *Mtb* Eis monomer and single DUSP16/MKP-7 with *Mtb* Eis dimer. The only condition we impose is an unambiguous distance requirement of 3.0 Å with a minimum 1.0 Å and a maximum 5.0 Å between the sulfur atom of acetyl CoA and the nitrogen atom of the Lys55 side-chain in DUSP16/MKP-7. In another trial, we imposed active (acetyl-CoA) and passive sites (Thr31, Asp32, Glu37, Arg43, Asp116, His125, Ser127, Glu128, Gly129, Tyr132, Arg134, Pro139, Leu143, Thr147, Val201, Lys211, Pro214, Thr237, Gln297, Trp301, Arg303, Met305, His318, Glu407 and Phe408) on *Mtb* Eis according to Kim *et al.* (2012 ▶) without distance restraints. For the *HADDOCK* passive site, we calculated the solvent accessibility of each residue using *MOLMOL* (Koradi *et al.*, 1996 ▶) and residues near the active site with solvent accessibility greater than 40% were taken. However, the results were essentially the same. Therefore, we imposed a distance requirement only in subsequent docking with the *Mtb* Eis dimer. As for DUSP16/MKP-7, we used Lys55 as the active site with no passive site. For the analysis of the docked structures, we have used root-mean-square-deviation-(RMSD)-based cluster analysis as incorporated in *HADDOCK* (Fig. 1 ▶). The structure figures were plotted using the program *PyMOL* (http://pymol.sourceforge.net).

## 3. Results and discussion

### 3.1. Docking

**Figure 2:**
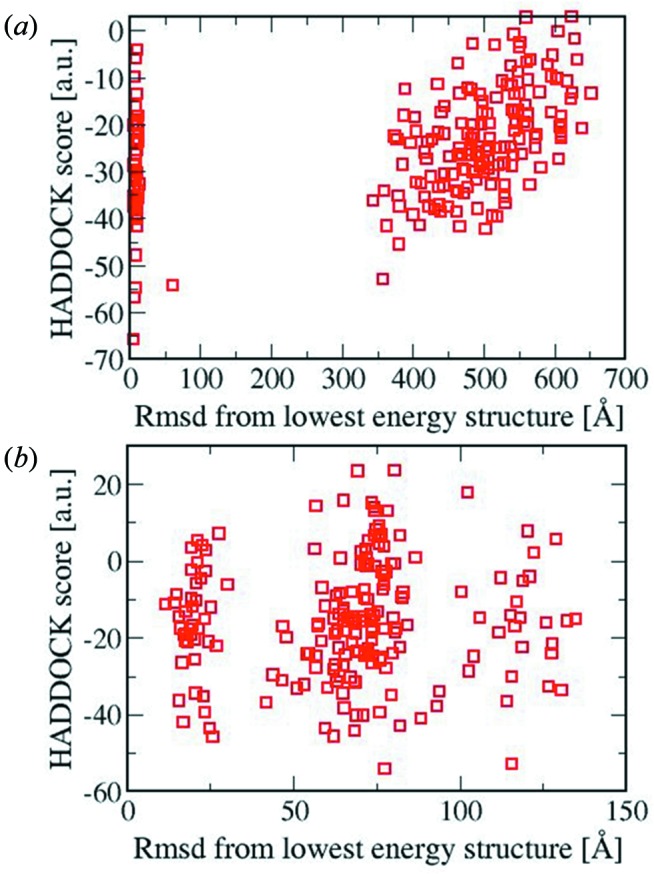
The RMSD *versus* *HADDOCK* score plot for the final 200 structures for the monomer (*a*) and dimer (*b*) of *Mtb* Eis. The reference structure for RMSD is the structure with the lowest *HADDOCK* score. The structures with RMSD > 50 Å do not meet the imposed distance restraint.

**Figure 3:**
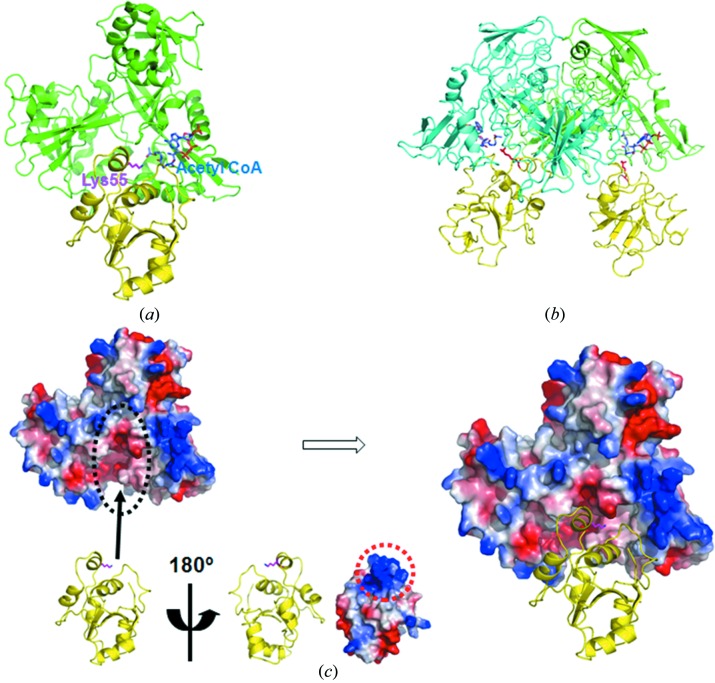
Docking model of the *Mtb* Eis monomer with DUSP16/MKP-7. (*a*) Cartoon docking model of *Mtb* Eis monomer (PDB code 3ryo: green) and DUSP16/MKP-7 (PDB code 2vsw: yellow). Acetyl-CoA (blue) bound to Eis and Lys55 (magenta) of DUSP16/MKP-7 are shown as a ball-and-stick model. (*b*) Cartoon docking model of *Mtb* Eis dimer (PDB code 3ryo: green and cyan) and DUSP16/MKP-7 (PDB code 2vsw: yellow). Acetyl-CoA (blue) bound to Eis and Lys55 (red) of DUSP16/MKP-7 are shown as a ball-and-stick model. (*c*) The same monomer docking model is presented as an electrostatic surface diagram of *Mtb* Eis and a cartoon model of DUSP16/MKP-7. A black dotted circle indicates the active binding site of *Mtb* Eis. Both the cartoon model and electrostatic surface diagram of DUSP16/MKP-7 rotated by 180° are shown on a smaller scale. A red dotted circle indicates the binding site of DUSP16/MKP-7 with positive electrostatic potential. Blue and red colors of the electrostatic surface diagrams correspond to positive and negative electrostatic potentials at neutral pH, respectively.

In the crystal lattice, *Mtb* Eis forms a hexamer of 32 symmetry, in which three dimeric units are related by threefold symmetry. The active site is formed within each monomer, well separated from the subunit interface. However, the active site is blocked in the hexamer assembly by adjacent subunits related by the threefold symmetry. In contrast, the active site is fully accessible in the dimer assembly just as in an isolated monomer. This observation suggests the possibility that Eis would exist, transiently, as a monomer or a dimer, rather than as a hexamer, in order to bind and acetylate the substrate DUSP16/MKP-7. The dimer would be a more plausible unit than the monomer, because further dissociation of a dimeric unit into monomers is expected to be energetically less favorable. However, prediction of dominant species by considering the entropy alone is not straightforward. We performed the docking for both the dimer and monomer of *Mtb* Eis; the active-site cleft of *Mtb* Eis is fully accessible to the substrate, DUSP16/MKP-7, in both cases. For each docking study we have 200 possible docked structures after the water solvation step, since we retain the best 50% from 400 refined structures. As for the monomer, from RMSD-based structural clustering analysis with RMSD criteria ranging from 4.0 to 10.0 Å, the number of clusters with more than a single member in its cluster is only one. The number of structures in this single cluster is 45 regardless of RMSD criteria. This indicates that the final docked structures are structurally very diverse except structures within this single cluster, which has minimal structural diversity between them. We plotted the RMSD *versus* *HADDOCK* score of these 200 structures in Fig. 2 ▶. In fact, only these 45 structures within the cluster satisfied the distance restraint that we imposed. We also note that the best *HADDOCK* scored structure belongs to this cluster. We have represented the best score monomer (*a*) and dimer (*b*) structures in Fig. 3 ▶ along with Lys55 in DUSP16/MKP-7 and acetyl-CoA.

Overall structures of both *Mtb* Eis and DUSP16/MKP-7 were maintained during the docking calculation. When we compare the initial structures of *Mtb* Eis (PDB ID 3ryo) and DUSP16/MKP-7 (PDB ID 2vsw) with those of *Mtb* Eis and DUSP16/MKP-7 from the best monomer *Mtb* Eis docking model, RMSDs are 1.19 Å and 1.15 Å, respectively. The largest Cα deviations occur at *Mtb* Eis Glu80 and Arg178, with 2.77 Å and 2.85 Å, respectively, and at DUSP16/MKP-7 Lys52 and Lys55, with 3.33 Å and 2.80 Å, respectively.

### 3.2. Structural features of the docking model

**Figure 4:**
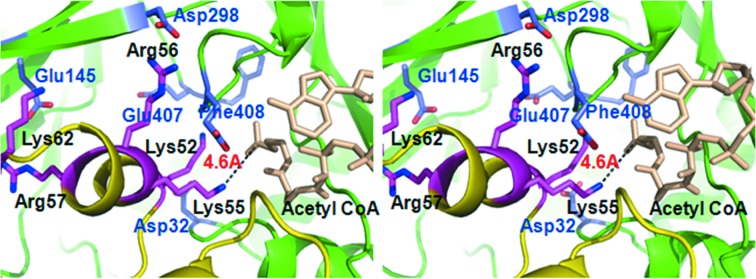
Enlarged stereoview of the active binding site in the docking model. Side-chains (in blue) and acetyl-CoA (in wheat color) of *Mtb* Eis (backbone in green and residues labeled in blue). Side-chains (in magenta) of DUSP16/MKP-7 (backbone in yellow and residues labeled in black) are shown as a ball-and-stick model.

Lys55 of DUSP16/MKP-7, the target of the acetylation by *Mtb* Eis, is included in the helix spanning residues from Lys52 to Gln60, which is referred to as the substrate helix hereafter in this paper. The key issue of this docking study should be how the substrate helix of DUSP16/MKP-7 fits into the active-site cleft of *Mtb* Eis, which was described previously as a ‘deep and narrow channel’ (Kim *et al.*, 2012 ▶), and thereby secures the structural environment for an acetylation reaction to occur. In our current docking model, the substrate helix fits nicely into the active-site cleft of *Mtb* Eis; the twisted β-sheet of Eis domain II embraces the substrate helix from one side [Figs. 3(*a*) and 3(*c*) ▶]. In particular, the side-chain of Lys55 is inserted toward acetyl-CoA, resulting in a distance of 4.6 Å between the NZ atom of Lys55 and the carbonyl carbon of the acetyl group (Fig. 4 ▶). The observed proximity here between the acetyl donor and the acceptor is close enough to fully facilitate the transfer of the acetyl group to occur.

Strikingly, the aforementioned structural fit between the active-site cleft of *Mtb* Eis and the substrate helix of DUSP16/MKP-7 is driven by favorable complementary charges at their interface. The active-site cleft of *Mtb* Eis has a negatively charged surface (Fig. 3*c* ▶) with Asp25, Glu138, Asp286, Glu395 and the terminal carboxylic group of Phe396 (Fig. 4 ▶), while the substrate helix of DUSP16/MKP-7 contains four basic residues, Lys52, Lys55, Arg56 and Arg57. In addition, the positively charged Lys62 is also located at the C-terminus of the helix (Fig. 4 ▶). All these five positively charged residues of the substrate helix of DUSP16/MKP-7 point toward the negatively charged surface of the active site of *Mtb* Eis. Acetylation is a crucial step for the enhanced survival capability of *Msm* against human autophagy. Understanding its action from a structural point of view is an important starting point for therapeutic purposes. The current docking model suggests that the binding of DUSP16/MKP-7 to *Mtb* Eis should be established by the charge complementarity, combined with favorable geometric shape complementarity between the substrate helix and the active-site cleft. We note that the present docking model requires the dissociation of hexameric *Mtb* Eis into dimers or monomers, at least transiently, for binding of DUSP16/MKP-7. Similar dissociation of other multimeric enzymes has been reported (Kernstock *et al.*, 2012 ▶). We could detect only hexameric species of *Mtb* Eis in the presence of DUSP16/MKP-7 (residues 1–153) by size-exclusion chromatography. This result may be expected, because the dissociation of *Mtb* Eis hexamers would be only transient, if it occurs. Therefore, we plan to produce a fusion protein between *Mtb* Eis and DUSP16/MKP-7 to test the possibility. By presenting a putative docked model, we hope that this study will provide a useful basis for future efforts to characterize in more detail the binding interface between *Mtb* Eis and DUSP16/MKP-7, and to develop inhibitors of *Mtb* Eis as a new tuberculosis drug candidate.
